# Editorial: Cancer Ecosystems

**DOI:** 10.3389/fonc.2019.00718

**Published:** 2019-08-20

**Authors:** Ubaldo E. Martinez-Outschoorn, Mireia Bartrons, Ramon Bartrons

**Affiliations:** ^1^Department of Medical Oncology, Sidney Kimmel Cancer Center, Thomas Jefferson University, Philadelphia, PA, United States; ^2^Aquatic Ecology Group, University of Vic - Central University of Catalonia, Vic, Spain; ^3^Unitat de Bioquímica, Departament de Ciències Fisiològiques, Universitat de Barcelona, Barcelona, Spain

**Keywords:** cancer ecosystem, transforming growth factor-β, mitochondrial transfer, tunneling nanotubes, fructose 2, 6-bisphosphate, monocarboxylate transporter 4

Oncology research pioneers such as Stephen Paget focused on how cancer cells favor particular environments (1) and Judah Folkman on how nutrients are provided to these harsh environments (2). The tumors consist of a heterogeneous population of cancer cells and a stroma with different cell types that define a specific microenvironment and form a tumoral ecosystem. The evolution of the tumors depends on the interactions of the cancer cells with their tumor microenvironment (TME), determining the progression, eradication, or tumor metastasis. A coral ecosystem is similar to tumors in that it is highly complex and energetically productive (3, 4) (Table 1). A tropical reef-building coral holobiont is composed of the coral metazoan host (the polyp), its endosymbiotic photosynthetic dinoflagellates (Symbiodiniaceae) and other microorganisms, including protozoans, fungi, bacteria, and archaea (5). Despite their complexity and very high productivity, corals commonly thrive in nutrient-poor environments (14), which are similar to what is observed in tumors. The contradiction of high coral productivity and limited nutrient availability has been named as the “Darwin Paradox,” in reference to its first discoverer (19). This paradox can be explained by the high uptake and efficient recycling of nutrients by coral reef organisms. A similar paradox has been observed in tumors since it is unclear how this complex ecosystem thrives in such nutrient deprived conditions (4).

**Table 1:** Examples of similarities between Tumor and Coral Reef Ecosystems.

**Tumor**	**Coral reef**
Highly complex ecosystems with high nutrient utilization rates (4)	Highly complex ecosystems with high nutrient utilization rates (3)
Nutrient deprivation: Compromised blood supply (Zuazo-Gaztelu and Casanovas)	Nutrient deprivation: Nitrogen and Phosphorus deprivation (5–7) Compromised water flow (8)
Metabolic coupling: Between cancer and stromal cells (9) Between cancer cells (10) Between cancer cell organelles (Herst et al.)	Metabolic coupling: Between coral and fish (11, 12) Between coral and sponges (13) Between coral and symbiodiniaceae (14)
Importance of mitochondria-endosymbiont in metabolic coupling (4)	Importance of photosynthetic bacteria in metabolic coupling (15)
Anticancer immunity (Fox et al.)	Immunity to maintain homeostasis (16)
Apoptosis (Leverson and Cojocari)	Apoptosis (17)
TGF-Beta (Fabregat and Caballero-Diaz)	TGF-Beta (18)

Scientists have debated how the extremely high productivity and diversity of coral reefs can thrive while living in such an oligotrophic environment, equivalent to a marine desert (13, 20). The answer relies on coral mutualistic relationships that allow the retention of resources and avoid their drift away in ocean currents. Sponges have been found to be the basis of this recycling of nutrients and energy back into the ecosystem (13). The largest resource produced on reefs are dissolved in organic matter (DOM), and sponges allow DOM to be transferred to higher trophic levels (13). In tumors, Otto Warburg focused on the idea that the key organic matter is glucose. He postulated that tumor cells maintain high glycolytic rates even with adequate oxygen supply, although he did not address how glucose becomes available to cancer cells (21). Compared to differentiated cells, many tumor cells have an altered energy metabolism. In particular, a change in metabolism based on respiration to one which is predominantly glycolytic (22, 23). Many glycolytic-related genes are systematically overexpressed in different types of cancer cells, have diagnostic utility, and may help predict therapeutic response (24). These data situate the glycolytic phenotype as a distinctive sign of tumor cells (22, 23, 25), providing advantages for proliferation.

Phosphofructokinase 1 (PFK1) and monocarboxylate transporter 4 (MCT4) are rate limiting steps in glycolysis (26). In this e-book collection, the role of one of the main glycolytic regulators, fructose 2,6-bisphosphate (Fru-2,6-P_2_), in cancer cells is reviewed by Bartrons et al. Fru-2,6-P_2_ allosterically induces PFK1 activity. TP53 Induced Glycolysis and Apoptosis Regulator (TIGAR) also regulates glycolysis via Fru-2,6-P_2_ and drives metabolic symbiosis between cancer cells and cancer-associated fibroblasts (27). Another key regulator of glycolysis: monocarboxylate transporter 4 (MCT4) is also studied by Bisetto et al. and found to induce tumor aggressiveness. MCT4 is the main exporter of lactate from cells, a marker of glycolysis and is regulated by HIF-1α. Conversely, MCT1 is the main importer of lactate into cells, a marker of mitochondrial oxidative phosphorylation and is regulated by c-MYC (4). Most complex ecosystems such as coral reefs and tumors have heterogeneous metabolic activity. Only some cells have high activity of a particular metabolic pathway. As an example, MCT1 is highly expressed in cancer cells while MCT1 expression is low or absent in tumor-associated macrophages (28).

Cancer cells and corals do not exist in isolation. In fact, all living entities host diverse symbionts that contribute to their associated functions. Most studies on the metabolism of cancer cells have focused on the investigation of a single type of intra-tumoral cell, although recent studies have described a more complex scene, where the tumor ecosystem via metabolic symbiosis plays a critical role in cancer progression (9, 10, 29). Similar metabolic interactions to those observed in tumors occur in corals. The “coral probiotic hypothesis” states that corals have a dynamic relationship with their symbiotic microorganisms. By altering the population of symbionts, the coral host adapts to a changing environment. This adjustment of a time span of days to weeks is faster than if it were via mutation and selection that would take many years (30). In sum, it is the combined holobiont that exerts the unit of natural selection as opposed to its individual members and it has been named the hologenome theory of evolution (31).

Cancer cells have a great capacity to adapt to changes in the conditions of their TME, developing survival strategies (Leverson and Cojocari). Similar plasticity has been observed in coral reefs (17). The tumor and stromal cells establish a powerful relationship that determines the initiation and progression of the disease, as well as the patient's prognosis (32). Physiologically, the stroma is a physical barrier against tumorigenesis; however, cancer cells elicit changes to convert the adjacent TME into a pathological entity, favoring nutrient exchange, migration of stromal cells, matrix remodeling, and expansion of the vasculature. In addition to malignant cells, the TME contains stromal cells that have been implicated in tumor promotion, such as endothelial cells of the blood and lymphatic circulatory system, pericytes, fibroblasts, and various bone marrow derived cells, including macrophages, neutrophils, mast cells, myeloid cell-derived suppressor cells, and mesenchymal stem cells, and sometimes even adipocytes (33). The Coral–Symbiodiniaceae relationship depends on nutrient interactions and metabolism between the coral host and the microbial symbionts in response to environmental conditions (34), sharing features with intercellular relationships in tumors. In fact, species richness is a key driver of community biomass production and ecosystem function across a range of ecosystems (35).

Fibroblasts in the TME can be activated. Here they are referred to as cancer-associated fibroblasts (CAFs), and they can be recruited to the tumor by different cytokines and growth factors released by cancer and infiltrated cells. Activation into CAFs is accomplished through genetic modifications and altered activation of different signaling pathways, such as NFκB, IL-6/STAT3, FGF-2/FGFR1, and TGF-β/SMAD (Herst et al.). Recent research, reviewed by Herst et al., shows that stromal cells have the ability to transfer mitochondria to tumor cells deficient in respiration, thus restoring mitochondrial respiratory capacity. Alterations in nuclear and mitochondrial DNA can affect mitochondrial respiration, forcing the cells to search for anaerobic pathways for obtaining energy. These cells with a predominantly glycolytic metabolism tend to have rapid growth, are resistant to hypoxia and can produce metastasis. In contrast, cells lacking mitochondrial DNA cannot form tumors unless they incorporate mitochondrial DNA from neighboring cells. These data suggest that mitochondrial exchange between cells could be used as an additional target in the treatment of cancer. In addition, cancer cells as well as different stromal cells can modify their metabolism in response to different signaling pathways, which can affect the therapeutic response. The resilience of coral reefs to global warming is also dependent on the crosstalk between different cells in this ecosystem (36). In this sense, the NFκB transcription factor, important in regulating cellular responses, is activated when elevated water temperature or other environmental perturbations induce the loss of the algal symbiont Symbiodinium (37) (Table 1).

The crosstalk between cancer cells and macrophages in the TME is investigated by Li et al., with the aim of study the intercellular communication in the tumor ecosystem. Identification of the different mechanisms of transport between the cells of the TME is essential to understand the mechanisms of tumor growth and is herein reviewed by Lou et al. These authors demonstrate that the intercellular exchange of microRNAs, mitochondria and other components is carried out through tunneling nanotubes (TNTs), cytoplasmic extensions based on actin. This transport through TNTs between malignant and stromal cells can modify the gene regulation and metabolism of TME cells, playing a critical role in tumor growth and metastasis, as well as in resistance to different treatments. Similarly, species particular trait values, such as fast growth rates and unique feeding strategies, can strongly affect ecosystem functions, such as coral reef productivity and nutrient cycling (38, 39). Alternatively, biodiversity can enhance ecosystem efficiency with a more complete utilization of resources (40–42). Such synergies are common in ecosystems like coral reefs, often occurring when functionally distinctive taxa increase the performance of other members of the ecosystem (43, 44) (Table 1).

The growth of tumor cells requires the supply of nutrients and oxygen. Therefore, the angiogenic program driven by TME cells is one of the first requirements in the tumoral ecosystem (2) and it is explored by Zuazo-Gaztelu and Casanovas. Angiogenesis, in addition to providing nutrients and oxygen, also facilitates the spread of tumor cells. Therefore, the blockade of this process has been proposed in the treatment of different types of cancer [(2); Zuazo-Gaztelu and Casanovas]. In this review, special emphasis is done on the interaction between tumor and stromal cells, suggesting that the molecular mechanisms of these interactions may be used for the development of new antiangiogenic agents. Coral reefs are also subjected to nutrient deprivation on the basis of changes in water flow (Table 1).

The protection offered by TME to tumor growth can be diminished by conditions such as chronic inflammation, with the subsequent release of cytokines and growth factors (45). In this way, chronic inflammation can trigger a response in which the proliferative signals induced by stromal cells are gradually enlarged. Fabregat and Caballero-Díaz review the role of TGF-β in hepatocarcinogenesis, considering that when chronic inflammation is established, inflammatory cells produce mediators, such as TGF-β, responsible for the activation of quiescent hepatic stellate cells to myofibroblasts (MFB), which mediate the synthesis of extracellular matrix proteins and are responsible of fibrogenesis. In parallel, TGF-β induces also changes in tumor cell characteristics, conferring migratory properties (Fabregat and Caballero-Díaz), as well as a glycolytic phenotype (46). TGF-β can also play important roles in the symbiotic or mutualistic relationships within coral reefs (Table 1).

Cancer cells near blood vessels grow at a higher rate, due to the high availability of nutrients and oxygen. Their energetic needs are supported by glycolysis and mitochondrial oxidative phosphorylation. In contrast, cells exposed to a microenvironment where nutrient and oxygen supplies are reduced, depend more on glycolysis which requires novel strategies to survive and proliferate. Thus, it is not surprising that these cells display higher grades of malignancy and chemo-resistance. Metabolic synergy between cancer and stroma cells is a driver of cancer aggressiveness and it has been shown that lactate is an essential metabolic intermediate between TME cells, fueling the oxidative metabolism of oxygenated tumor cells. In this way, a tumor symbiosis is produced by which the glycolytic and oxidative cells exchange metabolic substrates (4, 10). This metabolic compartmentalization allows for the exchange of metabolites between stroma and cancer cells, and this synergy is a result of differential expression of transporters and isoenzymes (10, 29). The article by Bisetto et al. studies the role of MCT4, an exporter of lactate, in head and neck squamous cell carcinoma (HNSCC) aggressiveness. It is demonstrated that MCT4 is a driver of aggressive cancer, it may be used as a diagnostic marker and its inhibitors could have therapeutic utility to prevent invasive HNSCC (Bisetto et al.).

We are beginning to understand the factors and pathways driving the glycolytic phenotype and metabolic reprogramming of tumor cells. Many genes are involved in this transformation, including RAS, TP53, HIF-1, and c-MYC. Although the change to the glycolytic phenotype is not an indispensable requirement for malignant transformation, the majority of studies indicate that it is an important phenomenon associated with survival advantage for the cells in the TME. In the review by Vaziri-Gohar et al., mutant KRAS is analyzed as an important player in the metabolic reprogramming of the pancreatic ductal adenocarcinoma cells (PDA). There is growing interest in the therapeutic exploitation of new metabolic inhibitors and in this review several clinical trials currently underway in patients with PDA are discussed.

A new and promising strategy for the cancer treatment uses mitochondria, since they are important in the regulation of metabolism and apoptosis. Cancer cell mitochondria exhibit multiple differential features with respect to that of normal cells. Among them, a stronger mitochondrial membrane potential that can allow the accumulation of cytotoxic cationic molecules within the cancer cells. González-Rubio et al. investigate the selective cytotoxic effect of cationic 10-N-nonyl acridine orange (NAO) on human lung carcinoma H520 cells. This compound is able to interfere with mitochondrial function and is a promising antitumor agent. In a similar way, mitochondrial anti-apoptotic proteins like BCL-2, BCL-XL, and MCL-1 are overexpressed in cancer cells (47) and offer a mechanism of survival and selective advantage in the nutrient deprived environment of tumors and are therefore attractive drug targets. Leverson and Cojocari review the literature on the BCL-2-inhibitor Venetoclax, approved for use in chronic lymphocytic leukemia and now being studied in a number of other hematologic malignancies. The results presented suggest that lymphoid microenvironments have a preponderant role in the sensitivity of cancer cells to Venetoclax (48). Scatena et al. identified new therapeutic targets that are relatively unique to cancer stem cells (CSCs). CSCs overexpress regulatory proteins of mitochondrial activity and their inhibition may represent a potentially new approach to eradicating CSCs. Different FDA-approved antibiotics, including Doxycycline, target mitochondria and the results obtained with this drug clearly show that it can selectively eradicate CSCs in breast cancer patients *in vivo* (Scatena et al.). Applying translational research, a clinical trial performed by Curry et al. used Metformin, an oral anti-diabetic drug that inhibits mitochondrial complex I, in patients with head and neck squamous cell carcinoma (HNSCC). Metformin resulted in an increase in cancer cell apoptosis and altered the cellular TME with an increased infiltrate of CD8+ Teff and FoxP3 Tregs at the invasive tumor margin of lymph nodes, suggesting an immunomodulatory effect in HNSCC. Furthermore, a review of Lee et al. on Metformin, as a treatment for endometrial cancer, presents the available clinical data and the molecular mechanisms by which it exerts its effects, focusing on how it may modify the TME. The multiple effects of metformin on the regulation of metabolism, as well as the changes produced in intercellular communication, make it a promising drug for the treatment of different types of cancer.

One of the important challenges in the treatment of cancer is the persistence of drug-resistant cell populations. Resistant subpopulations arise, among other factors, through modifications in the TME. The accumulation of extracellular fibrous proteins and the modification of the extracellular matrix are associated with tumor progression. Joyce et al. have studied how this TME affects the sensitivity of breast cancer cells to chemotherapeutic treatment. The cultured breast carcinoma cells showed a stromal-dependent response to Doxorubicin, suggesting that the conditions of the tumor microenvironment largely govern the response to drugs.

A study by Mojena et al. investigated the effect of a series of compounds derived from benzylamine/2-thiophenomethylamine (ethylamine) that showed antitumor activity on different melanoma tumor cell lines. These compounds develop a potent cytotoxic/antiproliferative activity in cells *in vitro* and in animal models of melanoma tumors, enhancing animal survival.

Rappaport and Waldman explore the cGMP signaling in the intestinal epithelium and the mechanisms by which it opposes intestinal injury. In colorectal tumors, the expression of the endogenous ligand of Guanylate cyclase C (GUCY2C) is lost and the reconstitution of GUCY2C signaling through the genetic or oral replacement of the ligand opposes tumorigenesis in mice. These results suggest that colorectal cancer may arise in a tumor microenvironment with a functional inactivation of GUCY2C.

In recent years we have seen the progress of immunological therapy against different types of cancers. A crucial aspect for its development has been its ability to reverse the immunosuppression induced by tumors. In this sense, the catabolic enzyme of tryptophan indoleamine 2,3-dioxygenase-1 (IDO1) has received great attention as a driver of tumor-mediated suppression. It has been shown that IDO1 is overexpressed in different human cancers and associated with an unfavorable prognosis. Fox et al. review the action of an inhibitor of IDO1, Indoximod, as a co-adjuvant of treatment in several types of tumors.

In conclusion, the current collection gathers researchers studying disparate fields of the cancer ecosystem to better understand how to target it. One must consider historically how only limited efforts have been devoted to study cancer ecosystems, which provide additional information to that obtained when one component is studied in isolation. Tumor ecosystems share productivity features and vulnerabilities not only with coral reefs but also with swamps (36, 49) and future studies will need to determine their similarities and differences with other physiological and pathological ecosystems. The analysis and understanding of natural ecosystems can facilitate new ways of cancer treatment. It may be that, as in the Indian proverb of blind men encountering different parts of an elephant, specialized researchers have seen only one aspect of tumor aggressiveness and determined its mechanisms accordingly.

## Author Contributions

This invited editorial was conceived by UM-O and RB, contributing equally to the writing. MB provided information in her areas of expertise.

### Conflict of Interest Statement

The authors declare that the research was conducted in the absence of any commercial or financial relationships that could be construed as a potential conflict of interest.
